# Deficiency of Calcium-Independent Phospholipase A2 Beta Induces Brain Iron Accumulation through Upregulation of Divalent Metal Transporter 1

**DOI:** 10.1371/journal.pone.0141629

**Published:** 2015-10-27

**Authors:** Goichi Beck, Koei Shinzawa, Hideki Hayakawa, Kousuke Baba, Toru Yasuda, Hisae Sumi-Akamaru, Yoshihide Tsujimoto, Hideki Mochizuki

**Affiliations:** 1 Department of Neurology, Osaka University Graduate School of Medicine, Suita, Osaka, Japan; 2 Department of Medical Genetics, Osaka University Graduate School of Medicine, Suita, Osaka, Japan; 3 Department of Human Genetics, National Center for Child Health and Development, Tokyo, Japan; 4 Osaka Medical Center for Cancer and Cardiovascular Diseases, Osaka, Japan; CINVESTAV-IPN, MEXICO

## Abstract

Mutations in *PLA2G6* have been proposed to be the cause of neurodegeneration with brain iron accumulation type 2. The present study aimed to clarify the mechanism underlying brain iron accumulation during the deficiency of calcium-independent phospholipase A2 beta (iPLA_2_β), which is encoded by the *PLA2G6* gene. Perl’s staining with diaminobenzidine enhancement was used to visualize brain iron accumulation. Western blotting was used to investigate the expression of molecules involved in iron homeostasis, including divalent metal transporter 1 (DMT1) and iron regulatory proteins (IRP1 and 2), in the brains of iPLA_2_β-knockout (KO) mice as well as in *PLA2G6*-knockdown (KD) SH-SY5Y human neuroblastoma cells. Furthermore, mitochondrial functions such as ATP production were examined. We have discovered for the first time that marked iron deposition was observed in the brains of iPLA_2_β-KO mice since the early clinical stages. DMT1 and IRP2 were markedly upregulated in all examined brain regions of aged iPLA_2_β-KO mice compared to age-matched wild-type control mice. Moreover, peroxidized lipids were increased in the brains of iPLA_2_β-KO mice. DMT1 and IRPs were significantly upregulated in *PLA2G6*-KD cells compared with cells treated with negative control siRNA. Degeneration of the mitochondrial inner membrane and decrease of ATP production were observed in *PLA2G6*-KD cells. These results suggest that the genetic ablation of iPLA_2_β increased iron uptake in the brain through the activation of IRP2 and upregulation of DMT1, which may be associated with mitochondrial dysfunction.

## Introduction

Phospholipase A_2_ (PLA_2_) enzymes catalyze the hydrolysis of membrane glycerophospholipids such as phosphatidylcholine via cleavage of the *sn-2* acyl chains to yield free fatty acids and lysophospholipids. These enzymes are classified into three types: secretory PLA_2_ (sPLA_2_) family, cytosolic PLA_2_ (cPLA_2_) family, and calcium-independent PLA_2_ (iPLA_2_) family [1]. Calcium-independent phospholipase A_2_β (iPLA_2_β), which is encoded by the *PLA2G6* gene, has several functions, including remodeling of membrane phospholipids [2], fatty acid oxidation [3], release of docosahexaenoic and arachidonic acids (DHA and AA, respectively) [4], cell growth and signaling [5], and cell death [6]. Furthermore, iPLA_2_β has been reported to exist in and protect mitochondria [7]; it plays an important role in acyl-decomposition in cardiolipin, which is specific to the mitochondrial inner membrane [8].

Neurodegeneration with brain iron accumulation (NBIA) is a group of disorders characterized by dystonia, parkinsonism and spasticity, and by iron accumulation in specific regions of the brain, predominantly in the basal ganglia [9]. It was reported that mutations in the *PLA2G6* gene are associated with two childhood neurologic disorders: infantile neuroaxonal dystrophy (INAD) and NBIA type 2 [10, 11, 12]. *PLA2G6*-associated neurodegeneration (PLAN) is the second core NBIA syndrome after pantothenate kinase-associated neurodegeneration (PKAN, formerly known as Hallervorden–Spatz disease) [13]. Previously, we and other groups demonstrated that iPLA_2_β-knockout (KO) mice showed slowly progressive motor deficits and tubulovesicular structures, which are neuropathologically specific for INAD [14, 15], suggesting that these mice would be a good animal model for INAD.

Iron homeostasis in the central nervous system (CNS) is mainly regulated by coordinated expression of molecules involved in iron uptake and storage. Initially, iron enters brain vascular endothelial cells (BVECs) through receptor-mediated endocytosis following interactions between transferrin receptor 1 (TfR1) and transferrin (Tf) [16]. Non-transferrin-bound iron (NTBI), released by BVECs, is quickly taken up by nearby astrocytes through divalent metal transporter 1 (DMT1) [17]. Iron is then transported into neurons, where it is an essential trophic factor required for oxygen consumption and ATP generation, through the Tf-TfR system and DMT1 [18]. Iron availability is regulated at the post-transcriptional level through interactions between iron-responsive elements (IREs) on mRNAs encoding proteins involved in iron metabolism, such as TfR1 and DMT1, and mRNA-binding proteins known as iron regulatory proteins (IRPs) [19]. Two IRPs (IRP1 and IRP2) are functionally similar cytosolic proteins that bind the same consensus IRE with equal affinity [20].

Mitochondria are subcellular organelles that are integral to all eukaryotic cells and are responsible for metabolic and respiratory functions. In addition to ATP production, mitochondria are the source of iron–sulfur clusters and heme [21]; it was reported that a rise in reactive oxygen species (ROS) and/or ATP depletion in mitochondria could augment the binding activity of IRPs [22, 23]. In this way, mitochondria have been positioned at the center of cellular iron homeostasis. Previous reports support the concept that mitochondrial dysfunctions play a crucial role in the pathogenesis of NBIA [21].

In the present study, for the first time we discovered that significant iron deposition was observed since early clinical stages in the brains of iPLA_2_β-KO mice compared to age-matched wild-type (WT) control mice. To clarify the mechanism underlying iron accumulation in the brains of iPLA_2_β-KO mice, we investigated the expression of molecules that are involved in iron homeostasis, including DMT1 and IRPs, in the brains of iPLA_2_β-KO mice as well as in *PLA2G6* knockdown (KD) SH-SY5Y human neuroblastoma cells. Mitochondrial function was also examined in *PLA2G6*-KD cells.

## Materials and Methods

### Animals

Mice with homozygous disruption of the iPLA_2_β gene on a C57BL/6 background [14], aged 15 weeks (*preclinical stage*, n = 2, 1 male and 1 female), 56 weeks (*early clinical stage*, n = 7, 3 males and 4 females), and 100 weeks (*late clinical stage*, n = 8, 5 males and 3 females), and wild-type (WT) mice aged 15 weeks (n = 3, all males), 56 weeks (n = 6, all males), and 100 weeks (n = 8, 4 males and 4 females) were used. After an overdose of isoflurane, each animal was perfused with phosphate buffered saline (PBS) followed by removal of the brains. To perform iron staining and immunohistochemistry for 4-hydroxy-2-nonenal (4-HNE), brains were immersed in 4% paraformaldehyde (PFA) overnight at 4°C, dehydrated, and embedded in paraffin blocks. Four-micrometer-thick paraffin sections were prepared. For free-floating immunohistochemistry, hemisphere brain blocks of mice were fixed overnight using 4% PFA in PBS and then immersed in PBS containing 30% sucrose until sinking. Coronal sections of the entire rostrocaudal extent of the substantia nigra (SN) were serially cut 20-μm thick using a cryostat (CM1850; Leica Microsystems). For Western blotting, brain blocks of mice perfused transcardially with PBS were dissected using the method described previously by our group [24] to yield striatum (ST) tissues, ventral parts of midbrain tissues, including the SN, cerebral cortex tissues, and cerebellum. After completing the dissection, the sections were immediately frozen on dry ice and stored at −80°C until analysis.

This study was carried out in strict accordance with the Guidelines for Animal Experimentation of the Japanese Association for Laboratory Animal Science. All animals were handled in accordance with the Guidelines for Animal Experimentation of Osaka University. The experimental protocol was approved by the Ethical Review Committee for Animal Experimentation of Osaka University School of Medicine (Permit Number: 26-044-000). All efforts were made to minimize suffering.

### Iron staining (diaminobenzidine enhancement of Perl’s staining)

Brain sections of iPLA_2_β-KO mice aged 15 weeks (n = 2, 1 male and 1 female), 56 weeks (n = 4, 4 females), and 100 weeks (n = 5, 2 males and 3 females), and WT mice aged 15 weeks (n = 3, all males), 56 weeks (n = 3, all males), and 100 weeks (n = 5, 1 male and 4 females) were used. Deparaffinized sections were incubated in Perl’s solution [1% K_4_Fe(CN)_6_ and 1% HCl] for 30 min, and then incubated in 0.05% diaminobenzidine (DAB, Vector Laboratories) for 15 min. Hydrogen peroxide (final concentration, 1%) was added, and samples were incubated for additional 30 min [25]. Hematoxylin was used to counterstain the cell nuclei.

Some of the specimens were immunostained with glial fibrillary acidic protein (GFAP) or ionized calcium binding adaptor molecule 1 (Iba-1) after iron staining. The primary antibodies used were a rabbit polyclonal antibody against GFAP (1:100, Dako) and a rabbit polyclonal antibody against Iba-1 (1:100, Wako). For the second immunostaining, VECTASTAIN ABC-AP kit (Vector Laboratories) and Alkaline Phosphatase Substrate Kit I VECTOR Red (Vector Laboratories) were used for the secondary antibody and for the visualization of reaction products, respectively.

### Free-floating immunohistochemistry and cell count

Brain tissues of iPLA_2_β-KO mice aged 56 weeks (n = 3, all males) and WT mice aged 56 weeks (n = 3, all males) were used. Free-floating sections were washed in PBS medium containing 0.05% Triton X-100 (PBS-T), and then incubated for 30 min with 0.3% H_2_O_2_ to quench endogenous peroxidase activity. The sections were soaked with blocking agents and then incubated with the primary antibodies dissolved in dilution reagent at 4°C for 24 h. Vector M.O.M. Immunodetection Kit (Vector Laboratories) was used for blocking and antibody dilution, according to the instructions provided by the manufacturer. The primary antibody used was a rabbit polyclonal antibody against tyrosine hydroxylase (TH, 1:1000, Calbiochem). Goat anti-rabbit immunoglobulins conjugated to peroxidase-labeled dextran polymer (Dako Envision+, Dako Corp.) were used as secondary antibodies. Reaction products were visualized using DAB (Vector Laboratories). The sections were mounted on glass slides and counterstained with cresyl violet (Nissl staining). The numbers of TH- and Nissl-double-positive cells in the SN pars compacta (SNpc) were counted in a blind manner in iPLA_2_β-KO mice and WT mice, as previously described [24]. SNpc cells that have nuclei optimally visible by TH immunostaining, and nuclei, cytoplasm, and nucleoli prominently stained by Nissl staining were counted. To avoid double counting of neurons with unusual shapes, TH- and Nissl-double-positive cells were counted only when their nuclei and nucleoli were optimally visualized. The rostral end of the SNpc was defined as the level where TH- and Nissl-double-positive cells began to appear, and the caudal end of the SNpc was defined as the level where TH- and Nissl-double-positive cells and oculomotor nerves could be observed [24]. The data were compared statistically by the Wilcoxon rank sum test (EXCEL Toukei ver. 6.0, ESUMI). A probability of p < 0.05 was considered statistically significant.

### Immunohistochemistry for 4-HNE

Brain tissues of iPLA_2_β-KO mice aged 100 weeks (n = 5, 2 males and 3 females) and WT mice aged 100 weeks (n = 5, 1 male and 4 females) were used. Deparaffinized sections were incubated for 30 min with 0.3% H_2_O_2_ to quench endogenous peroxidase activity and then washed with PBS. The primary antibody used was a mouse monoclonal antibody against 4-HNE (an oxidized secondary product that is formed when organic lipids consisting of polyunsaturated fatty acid (PUFA) receive oxidization stress; 1:100; NOF Corporation). It is known that 4-HNE forms a relatively stable reactant (Michael adduct) with proteins. The antibody used in this study is highly specific to the stable 4-HNE-protein compound. Autoclave treatment was performed for 9 min before incubation. Goat anti-mouse immunoglobulin (Ig) conjugated to peroxidase-labeled dextran polymer (Dako Envision+; Dako) was used as the secondary antibody. Reaction products were visualized using DAB (Vector Laboratories), and hematoxylin was used to counterstain the cell nuclei.

### Cell Culture

The SH-SY5Y neuroblastoma cell line was obtained from American Tissue Culture Collection (ATCC) and used within 20 passages of the original vial. Cells were grown in Dulbecco's modified Eagle's medium (DMEM, high-glucose formulation, Nacalai Tesque) supplemented with 10% fetal bovine serum (FBS, Gibco), 100 units/ml penicillin, and 100 μg/ml streptomycin. Cell cultures were all kept at 37°C in a saturated humidity air atmosphere containing 5% CO_2_.

### Transfection of siRNA

Small interfering RNA (siRNA) targeting the human *PLA2G6* gene and the negative control siRNA were purchased from Life technologies and Qiagen, respectively. Subconfluent SH-SY5Y cells were transfected with siRNAs using Lipofectamine RNAiMax (Invitrogen) every 24 h for a total of three times, according to the manufacturer's instructions. The targeting sense sequence for human *PLA2G6* in SH-SY5Y cells is 5′-GACCAAAGAGCAAGUGACAAAUGUU-3′.

### Reverse transcription polymerase chain reaction

Total RNA was extracted from siRNA-transfected SH-SY5Y cells using the RNeasy Kit (Qiagen), according to the manufacturer's instructions, and the RNA concentrations were determined spectrophotometrically. cDNA was synthesized from 5 μg total RNA using the SuperScript III reverse transcriptase and oligo dT (Invitrogen). cDNA was amplified by PCR (94°C for 3 min and 28 cycles of 94°C for 30 s, 55°C for 30 s, and 72°C for 1 min). Primers are listed as follows: sense primer for *PLA2G6* 5′-TGTCGAAAGACAACGTGGAGATGATCAAGG-3′, antisense primer 5′-GTTTCTGGAGCATCGTAGTTCCGGAAGAGG-3′. Amplified cDNA length of *PLA2G6* is 748 bp. β-actin was used as the endogenous control.

### Immunocytochemistry

Cells were rinsed with pre-warmed PBS (pH 7.2) and fixed in 4% PFA for 30 min. After washing with PBS three times, cells were permeabilized with 0.2% Triton X-100 for 30 min, and then incubated with 10% skim milk in PBS for 60 min. The primary antibodies used were a rabbit polyclonal antibody against the 20-kDa translocase of outer mitochondrial membranes (Tom20, 1:100, Santa-Cruz) and a mouse monoclonal antibody against cytochrome *c* oxidase (CCO, 1:100, Invitrogen). Alexa Fluor® 488 goat anti-rabbit IgG (H + L) antibody (Life Technologies) and Alexa Fluor® 568 goat anti-mouse IgG (H + L) antibody (Life Technologies) were used as the secondary antibodies. Each aforementioned step was performed at room temperature. Confocal laser-scanned images were obtained using an LSM 510 META (Carl Zeiss).

### Western blotting

Brain tissues of iPLA_2_β-KO mice aged 100 weeks (n = 3, all males) and WT mice aged 100 weeks (n = 3, all males) were used. Frozen tissues were sonicated in chilled CelLytic-MT mammalian tissue lysis/extraction reagent (Sigma-Aldrich) mixed with protease inhibitor mixture set I (Calbiochem) and phosphatase inhibitor mixture set V (Calbiochem). The samples were centrifuged (20,000 × g for 10 min at 4°C), and the resulting supernatants were collected for use. siRNA-transfected SH-SY5Y cells were collected after transfection for 48 h. Cells were directly lysed in SDS sample buffer (63 mM Tris–HCl, pH 6.8; 2% SDS; 5% sucrose; 5% 2-mercaptoethanol) and were boiled for 5 min. Protein concentrations were determined by Lowry method.

Proteins (10 μg for animal tissues and 2 μg for cells) were separated on 15% sodium dodecyl sulfate polyacrylamide gel electrophoresis (SDS-PAGE), electrotransferred to a polyvinylidene difluoride (PVDF) membrane (Bio-Rad), blocked with 5% nonfat milk in TBS–Tween buffer for 60 min at room temperature, and incubated overnight at 4°C with the primary antibodies. The primary antibodies used were a rabbit polyclonal antibody against TfR1 (Abcam, 1:500), a mouse monoclonal antibody against DMT1 + IRE (Abcam, 1:500), a rabbit polyclonal antibody against IRP1 (Novus, 1:500), a mouse monoclonal antibody against IRP2 (Abcam, 1:1000), a rabbit polyclonal antibody against Ferroportin 1 (FPN1, also known as solute carrier family 40 member 1, SLC40A1, Abcam 1:1000), a mouse monoclonal antibody against 4-HNE (NOF Corporation, 1:1000), a rabbit polyclonal antibody against Tom20 (1:500, Santa-Cruz), a mouse monoclonal antibody against CCO (1:500, Invitrogen), and a mouse monoclonal antibody against glyceraldehyde-3-phosphate dehydrogenase (GAPDH, Millipore, 1:1000). The membrane was washed 3 times with TBS–Tween buffer for 30 min (10 min per wash), and then incubated for 60 min in horseradish peroxidase-conjugated goat anti-mouse or anti-rabbit IgG at room temperature. After extensive washing, the bands were visualized with enhanced chemiluminescence's reagents (ECL Prime Western Blotting Detection System, GE Healthcare) and exposed to X-ray film. The densitometry of the bands was quantified using computer-assisted image analysis techniques (Image J, National Institutes of Health). The data were compared statistically by the Wilcoxon rank sum test (EXCEL Toukei ver. 6.0, ESUMI). A probability of p < 0.05 was taken to indicate statistical significance.

### Adenosine triphosphate (ATP) assay

siRNA-transfected SH-SY5Y cells were harvested by adding a solution of 1 mM EDTA in PBS, and then centrifuged at 50,000 rpm at 4°C for 5 min. After removing the supernatant, cells were frozen quickly at −80°C. After determination of protein concentrations by Lowry method, a Kinshiro ATP luminescence kit (LL100-1, Toyo INK) was used to determine the amount of ATP, according to the manufacturer's instructions. The data were compared statistically by the Wilcoxon rank sum test (EXCEL Toukei ver. 6.0, ESUMI), and a probability of p < 0.05 was taken to indicate statistical significance.

### Cell Titer Blue assay

The Cell Titer Blue® (CTB) assay (Promega) is based on the ability of living cells to convert a redox dye (resazurin) into a fluorescent end product (resorufin). Nonviable cells rapidly lose metabolic capacity and do not reduce the indicator dye; therefore, do not generate a fluorescent signal. Reagent is added directly to each well, and the plates are incubated at 37°C for 3 h. The fluorescence of each well was determined using a microplate reader (CytoFluor MultiWell Plate Reader Series 4000, PerSeptive Biosystems) at a wavelength of 530/620 nm to quantify cell viability.

### Lactate Dehydrogenase assay

Lactate dehydrogenase (LDH) Cytotoxicity Assay Kit (Roche Applied Science) was used to measure LDH released from the cells. The assay was performed using the manufacturer’s instructions. The absorbance of each sample at a wavelength of 492/620 nm was measured using a microplate reader (Microplate Reader SH-9000Lab, Corona Electric) and used to quantify cell viability.

## Results

### Age-dependent iron accumulation in the brains of iPLA_2_β-KO mice

**Fig 1:**
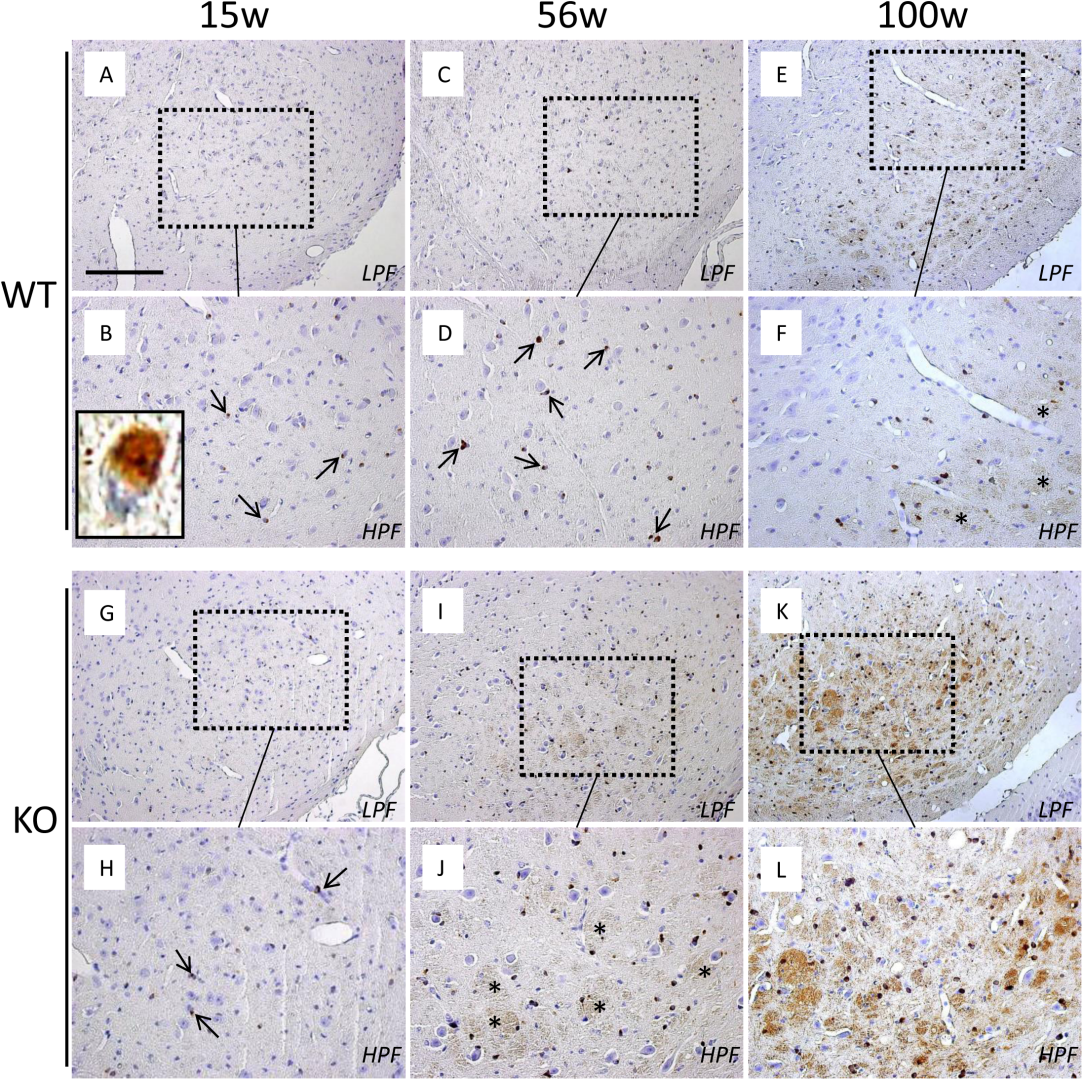
Age-dependent iron accumulation in the SN of iPLA_2_β-KO mice shown by Perl’s DAB enhanced staining. (A–L), Perl’s DAB enhanced staining of the SN of WT mice at 15 weeks (A and B), 56 weeks (C and D), and 100 weeks (E and F), and iPLA_2_β-KO mice at 15 weeks (G and H), 56 weeks (I and J), and 100 weeks (K and L). (B), (D), (F), (H), (J), and (L) are the high magnification views of (A), (C), (E), (G), (I), and (K), respectively (low power field, LPF; high power field, HPF). (A–F) In WT mice at 15 weeks, only a few iron depositions are observed mainly in oligodendrocytes (A, small arrows in B). The inset in (B) is a high magnification of an iron-containing oligodendrocyte. The number of iron deposition increases in WT at 56 weeks (C, small arrows in D), compared with that at 15 weeks (A and B). In WT mice at 100 weeks, iron depositions become more prominent mainly in SN pars reticulate and are observed in nerve fibers (asterisks in F) as well as oligodendrocytes (E and F), in comparison with those of WT mice at 56 weeks (C and D). (G–L) In iPLA_2_β-KO mice at 15 weeks, iron depositions are observed in a few oligodendrocytes (G, small arrows in H), which are almost equal with those of WT mice at 15 weeks (A and B). In iPLA_2_β-KO mice at 56 weeks, iron depositions significantly increase and are also observed in the nerve fibers (I, asterisks in J) compared with those of WT mice at 56 weeks (C and D). In KO mice at 100 weeks, marked iron depositions are observed in a large number of oligodendrocytes and nerve fibers (K and L), which are more prominent than iPLA_2_β-KO mice at 56 weeks (I and J) and WT mice at 100 weeks (E and F). Scale bar in (A) represents 100 μm in (A), (C), (E), (G), (I), and (K), and 50 μm in (B), (D), (F), (H), (J), and (L).

**Fig 2:**
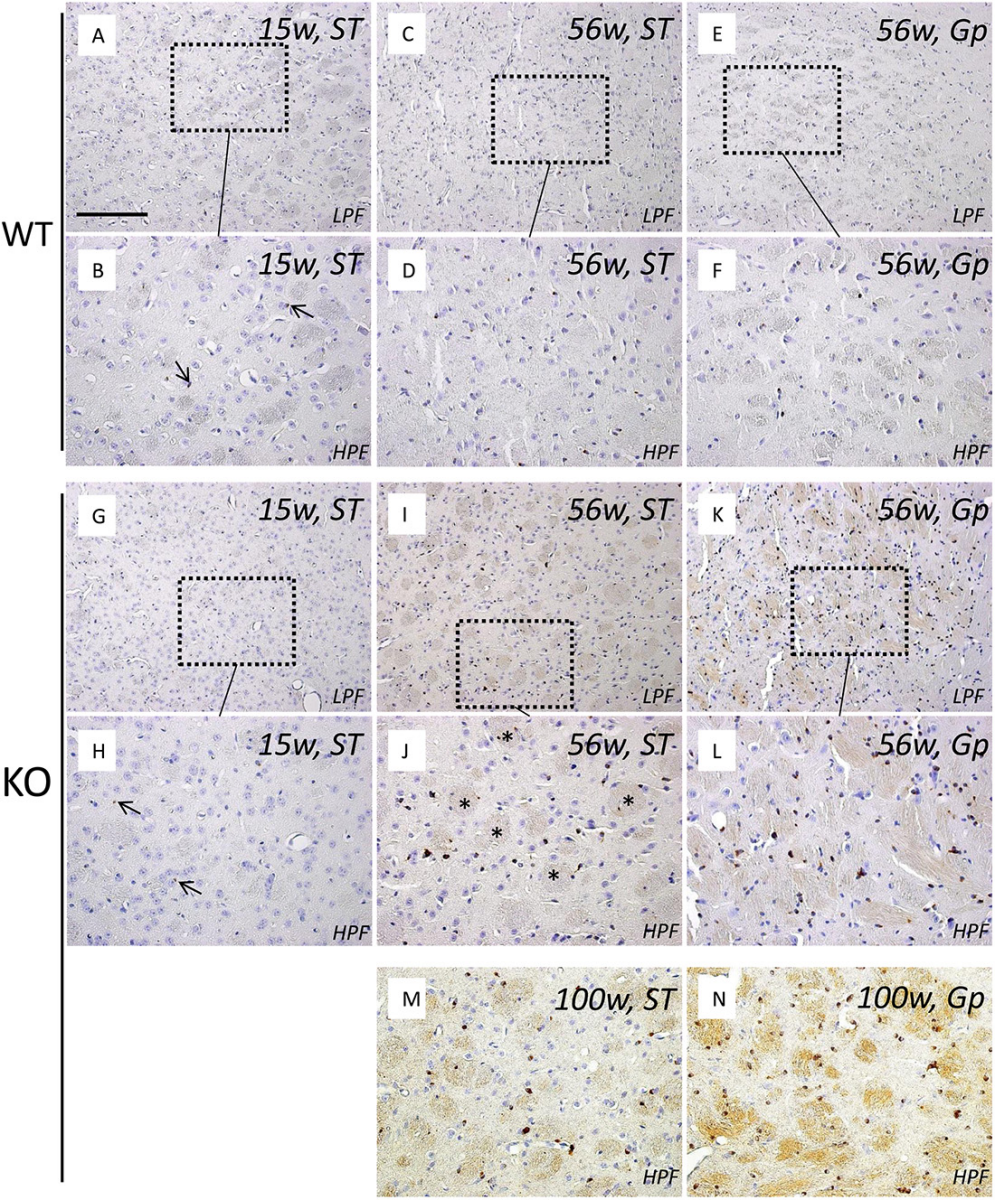
Age-dependent iron accumulation in other brain regions of iPLA_2_β-KO mice. (A‒N) Perl’s DAB enhanced staining of the ST of WT mice at 15 weeks (A and B) and 56 weeks (C and D); the Gp of WT mice at 56 weeks (E and F); the ST of iPLA_2_β-KO mice at 15 weeks (G and H), 56 weeks (I, J), and 100 weeks (M); the Gp of iPLA_2_β-KO mice at 56 weeks (K and L) and 100 weeks (N). (B), (D), (F), (H), (J), and (L) are high magnification views of (A), (C), (E), (G), (I), and (K), respectively (low power field, LPF; high power field, HPF). In the ST of WT mice at 15 weeks (A and B) and iPLA_2_β-KO mice at 15 weeks (G and H), only a few iron depositions are observed in oligodendrocytes (small arrows in B and H), which show no significant differences between them. In the ST of iPLA_2_β-KO mice at 56 weeks, iron depositions increased significantly and are also observed in the nerve fibers (I, asterisks in J), in comparison with those of WT mice at 56 weeks (C and D) and those of iPLA_2_β-KO mice at 15 weeks (G and H). In the Gp of iPLA_2_β-KO mice at 56 weeks, prominent iron depositions are seen in a lot of oligodendrocytes as well as nerve fibers (K and L), in comparison with those in the Gp of WT mice at 56 weeks (E and F). Iron accumulation becomes more prominent in the ST (M) and the Gp (N) of iPLA_2_β-KO mice at 100 weeks than those in the ST and Gp of iPLA_2_β-KO mice at 56 weeks (I, J, K, and L). Scale bar in (A) represents 100 μm in (A), (C), (E), (G), (I), (K), 50 μm in (B), (D), (F), (H), (J), (L), (M), (N).

DAB enhancement of Perl’s staining can visualize both ferrous iron (Fe^3+^) and ferric iron (Fe^2+^) [26]. In WT control mice at 15 weeks, only a few iron depositions were observed in some brain areas such as the substantia nigra pars reticulata (SNpr) (Fig 1A and 1B), the striatum (ST) (Fig 2A and 2B), and the globus pallidus (Gp) (data not shown). In these areas, iron was localized mainly in oligodendrocytes and in the fibrous network of the neuropil [27]. By double immunohistochemistry, these iron-containing glial cells were rarely positive for GFAP or Iba-1 (S1 Fig). These iron depositions increased with age. In WT mice at 100 weeks (Fig 1E and 1F), glial cells and fibers in the SNpr were positive for iron staining more significantly than those in WT mice at 15 weeks (Fig 1A and 1B) and 56 weeks (Fig 1C and 1D).

In iPLA_2_β-KO mice at 15 weeks (*preclinical stage*), iron depositions were observed in a few glial cells in the SNpr (Fig 1G and 1H) and the ST (Fig 2G and 2H), which were equivalent to those observe in WT mice at 15 weeks (Figs 1A, 1B, 2A and 2B). In iPLA_2_β-KO mice at 56 weeks (*early clinical stage*), iron depositions increased and were observed in the nerve fibers in the SNpr (Fig 1I and 1J) and the ST (Fig 2I and 2J), which showed a significant difference in comparison with those in WT mice at 56 weeks (Figs 1C, 1D, 2C and 2D). Particularly, in the Gp, iron depositions were found more prominently in KO mice at 56 weeks (Fig 2K and 2L) than those of WT mice at 56 weeks (Fig 2E and 2F). In iPLA_2_β-KO mice at 100 weeks (*late clinical stage*), significant iron depositions were observed in many glial cells and nerve fibers in the SNpr (Fig 1K and 1L), ST (Fig 2M), and Gp (Fig 2N), which were more prominent than those observed in iPLA_2_β-KO mice at 56 weeks (Figs 1I, 1J, 2I, 2J, 2K and 2L) and WT mice at 100 weeks (Fig 1E, 1F and S2 Fig). In KO mice at 100 weeks, iron depositions were also observed in some neurons of the cerebellum and in neurons of the hippocampus (S3 Fig).

### Increased brain expression levels of DMT1 and TfR1 of iPLA_2_β-KO mice

**Fig 3:**
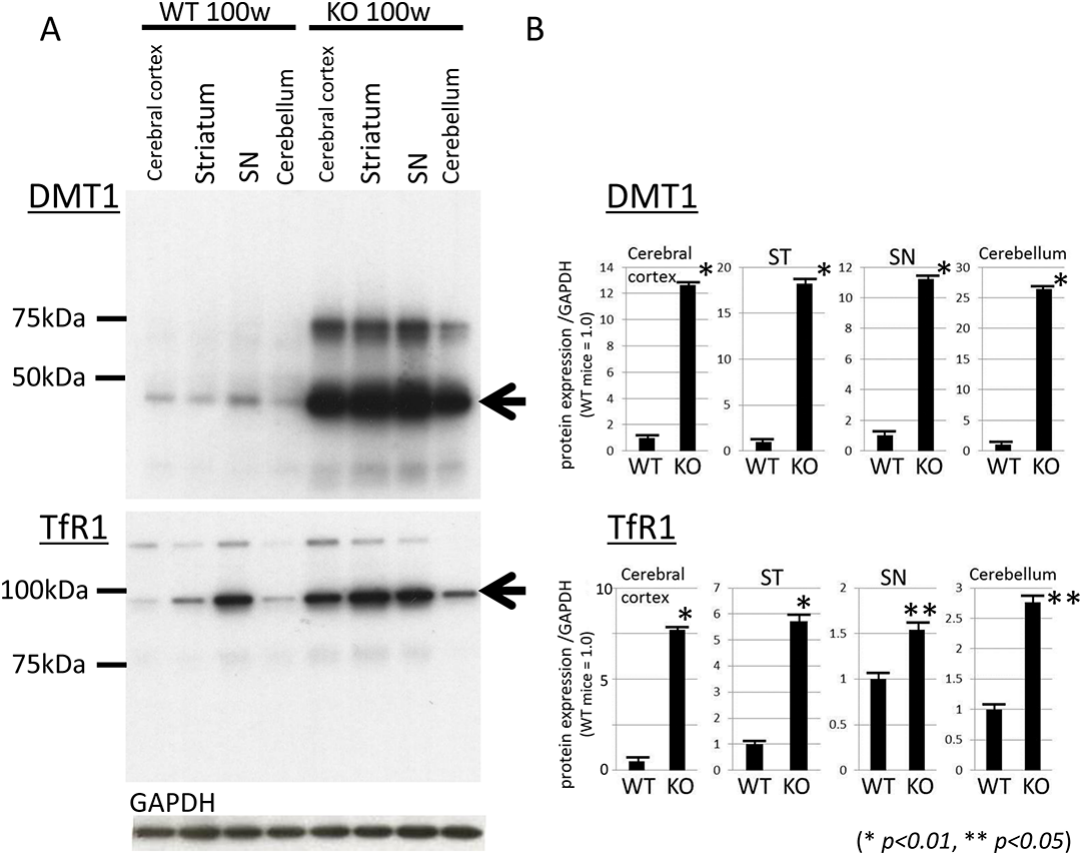
Western blotting analyses of DMT1 and TfR1 in iPLA_2_β-KO mice at 100 weeks. (A) Western blotting was applied to detect the expressions of DMT1 + IRE and TfR1 in iPLA_2_β-KO mice and WT mice at 100 weeks. (B) Statistical analysis. Data are presented as the ratio of TfR1 or DMT1 to GAPDH (WT mice = 1.0). Each bar represents the mean ± SD. *p < 0.*01*, **p < 0.*05*, Wilcoxon's rank-sum test.

We examined the expression levels of DMT1 with IRE (DMT1 + IRE) and TfR1 in the cerebral cortex, ST, SN, and cerebellum of WT and iPLA_2_β-KO mice at 100 weeks by Western blotting. We found that DMT1 + IRE significantly increased in all examined brain regions in iPLA_2_β-KO mice at 100 weeks, compared with those of WT mice at 100 weeks as seen in Fig 3A (with statistical significance [*p < 0*.*01*, Wilcoxon's rank sum test, Fig 3B]). Similarly, the increase of TfR1 was also found in the cerebral cortex, ST, and cerebellum of iPLA_2_β-KO mice at 100 weeks compared to age-matched WT mice as seen in Fig 3A (with statistical significance [*p < 0*.*01* or *p < 0*.*05*, Wilcoxon's rank sum test, Fig 3B]). In the SN of iPLA_2_β-KO mice at 100 weeks, the increase of TfR1 expression was the least prominent among those of other brain areas examined (Fig 3A), but showed statistical significance in comparison with that in the SN of WT mice at 100 weeks (*p < 0*.*05*, Wilcoxon's rank sum test, Fig 3B). The expression levels of DMT1 showed about 11- to 26-fold increase in the brains of iPLA_2_β-KO mice relative to those of WT mice (Fig 3B), whereas the expression levels of TfR1 showed about 1.5- to 7.5-fold increase in iPLA_2_β-KO mice (Fig 3B). At the same time, the expression levels of FPN1, the cellular iron exporter, were decreased in the brains of iPLA_2_β-KO mice at 100 weeks compared to age-matched WT mice (with statistical significance [*p < 0*.*05*, Wilcoxon's rank sum test], S4 Fig).

### Increased brain expression levels of IRPs in iPLA_2_β-KO mice

**Fig 4:**
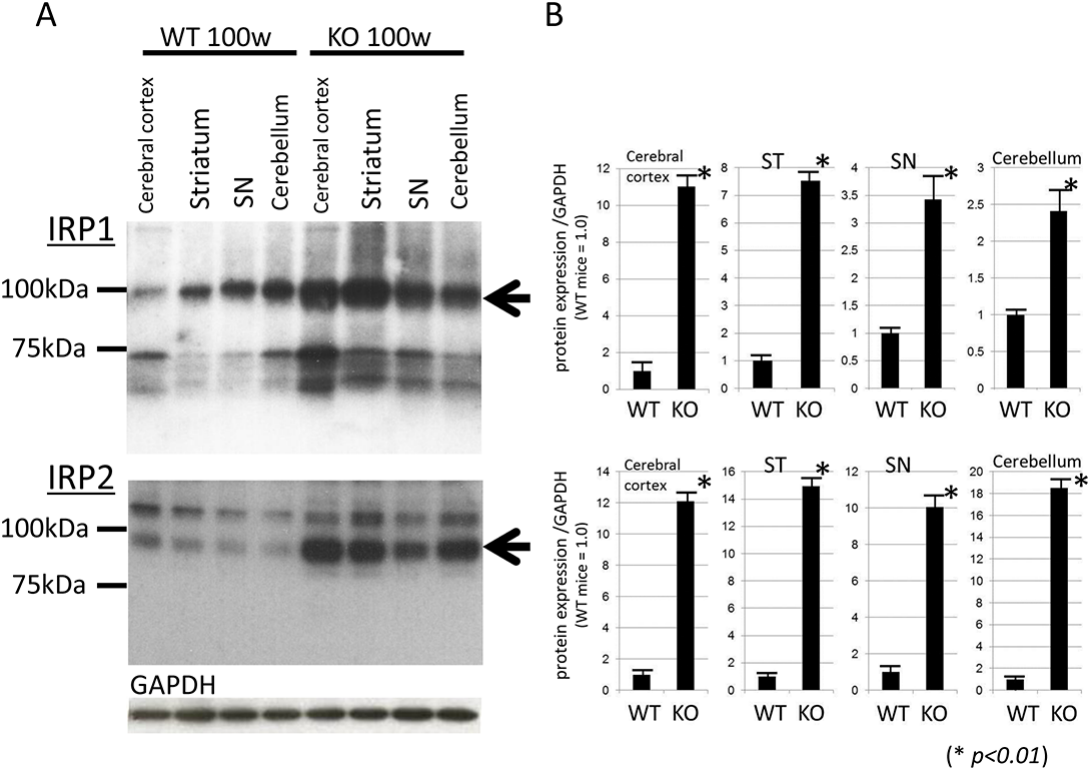
Western blotting analyses of IRPs in iPLA_2_β-KO mice at 100 weeks. (A) Western blotting was applied to detect the expressions of IRP1 and IRP2 in iPLA_2_β-KO mice and WT mice at 100 weeks. (B) Statistical analysis. Data are presented as the ratio of IRP1 or IRP2 to GAPDH (WT mice = 1.0). Each bar represents the mean ± SD. *p < 0.*01*, Wilcoxon's rank-sum test.

We examined the expression levels of IRP1 and IRP2 in the cerebral cortex, ST, SN, and cerebellum of WT and iPLA_2_β-KO mice at 100 weeks by Western blotting. The expression levels of IRP2 were prominently increased in all examined brain regions of iPLA_2_β-KO mice at 100 weeks compared with WT mice at 100 weeks (*p < 0*.*01*, Wilcoxon's rank sum test, Fig 4), and to a lesser extent, the expression levels of IRP1 also showed increases in the brains of iPLA_2_β-KO mice relative to those of WT mice with statistical significance (*p < 0*.*01*, Wilcoxon's rank sum test, Fig 4).

### Increase of peroxidized lipids in aged iPLA_2_β-KO mice

**Fig 5:**
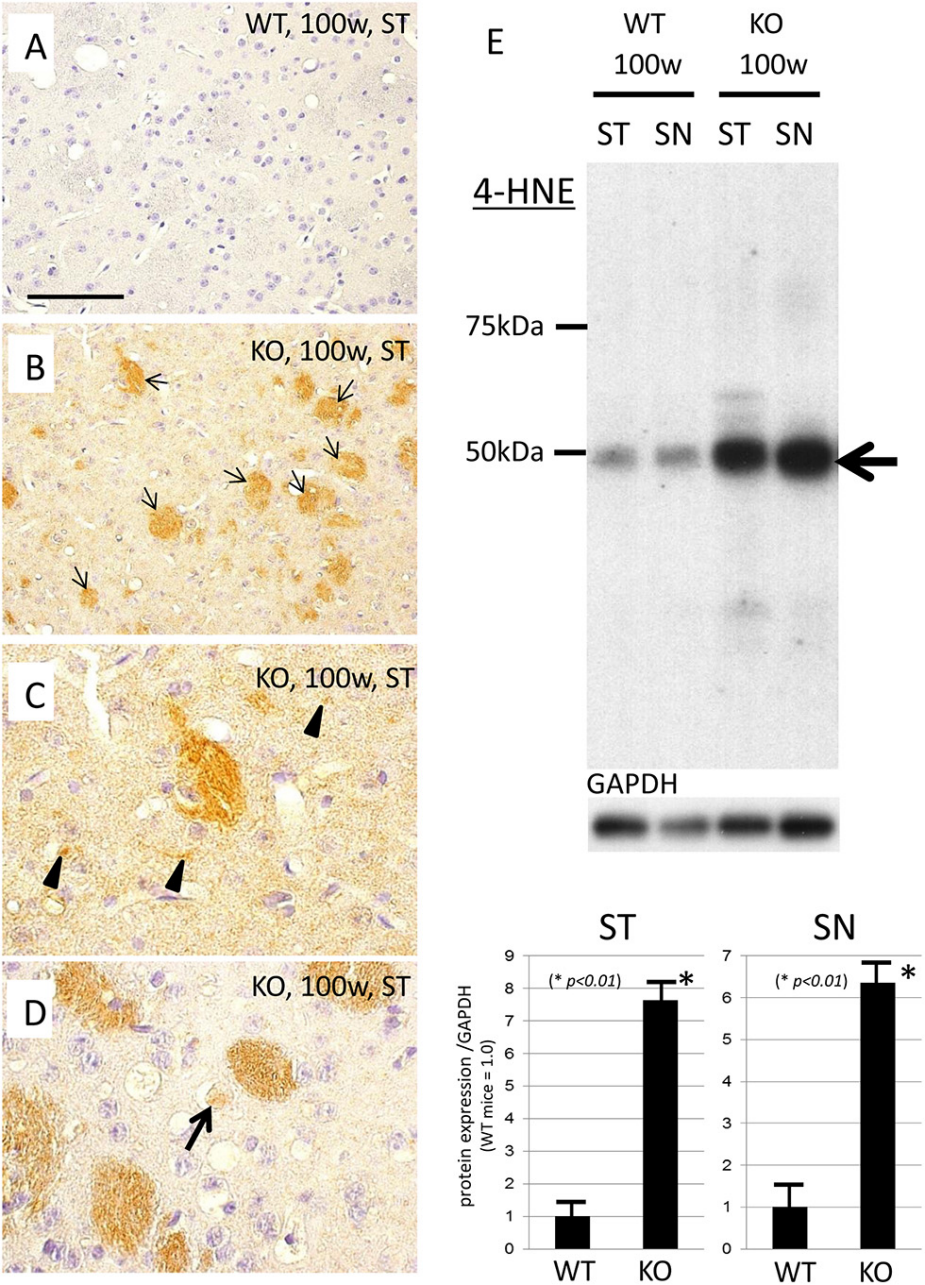
Immunohistochemistry and Western blotting analyses for 4-HNE in iPLA_2_β-KO mice. (A‒D) Immunohistochemistry for 4-HNE in the ST of WT mice (A) and iPLA_2_β-KO mice at 100 weeks (B‒D). (A) Almost no staining is observed in the ST of WT mice at 100 weeks. (B) In the ST of iPLA_2_β-KO mice at 100 weeks, the increase of 4-HNE is observed mainly in the white matter (small arrows), and the neuropil is also slightly immunostained with 4-HNE, compared with age-matched WT mice (A). (C, D) In high-power fields, some granules or dots strongly positive for 4-HNE are frequently observed (arrowheads in C) in the neuropil of the ST of iPLA_2_β-KO mice at 100 weeks. A few spheroids are also faintly immunopositive for 4-HNE (arrow in D). Scale bar in (A) represents 100 μm in (A), (B), and 50 μm in (C), (D), respectively. (E) On Western blotting, expression levels of 4-HNE-protein compound are increased in both ST and SN in iPLA_2_β-KO mice at 100 weeks in comparison with those of age-matched WT mice, with statistical significance (*p < 0.*01*, Wilcoxon's rank sum test). Data are presented as the ratio of 4-HNE-protein compound to GAPDH (WT mice = 1.0). Each bar represents the mean ± SD.

On immunohistochemistry for 4-HNE, minimal staining was observed in the ST of WT mice at 100 weeks (Fig 5A), whereas the increase in 4-HNE was observed mainly in the white matter in the ST of iPLA_2_β-KO mice at 100 weeks (Fig 5B–5D). The neuropils were also slightly immunostained with 4-HNE (Fig 5B–5D). Under high magnification, some granules or dots that were strongly positive for 4-HNE were frequently observed in the neuropils of the ST in iPLA_2_β-KO mice at 100 weeks (Fig 5C). A few spheroids that were slightly immunopositive for 4-HNE were also observed in the ST of iPLA_2_β-KO mice at 100 weeks (Fig 5D). As shown by Western blotting, the expression levels of 4-HNE-protein compound in the ST as well as SN were significantly increased in iPLA_2_β-KO mice at 100 weeks in comparison with age-matched WT mice (*p < 0*.*01*, Wilcoxon's rank sum test, Fig 5E).

### No evidence of dopaminergic cell loss was observed in iPLA_2_β-KO mice at 56 weeks

**Fig 6:**
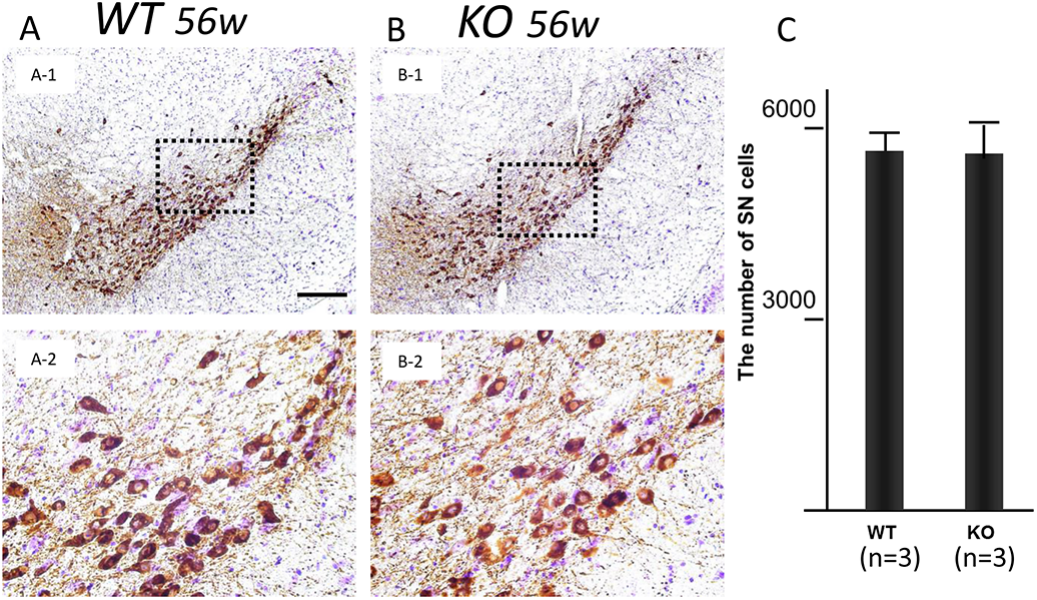
Dopamine cell counts in the SNpc in WT control mice and iPLA_2_β-KO mice. (A, B) Representative sections of SNpc immunostained with TH in WT (A) and KO mice (B). Panels A-2 and B-2 show high magnifications of dotted squares in A-1 and B-1, respectively. Scale bar in A-1 represents 100 μm in A-1 and B-1, and 25 μm in A-2 and B-2. (C) Histogram of the number of TH- and Nissl-double-positive neurons in the SNpc in WT (n = 3) and KO mice (n = 3) is shown. There is no significant difference between the two groups of mice (*p > 0*.*05*, Wilcoxon's rank sum test).

We then investigated the number of dopaminergic cells in the SNpc in WT (n = 3) and iPLA_2_β-KO mice (n = 3) at 56 weeks. There were no significant differences in the number of TH- and Nissl-double-positive cells in the SNpc between WT and iPLA_2_β-KO mice (*p > 0*.*05*, Wilcoxon's rank sum test) (Fig 6).

### Analysis of *PLA2G6*-KD SH-SY5Y cells

**Fig 7:**
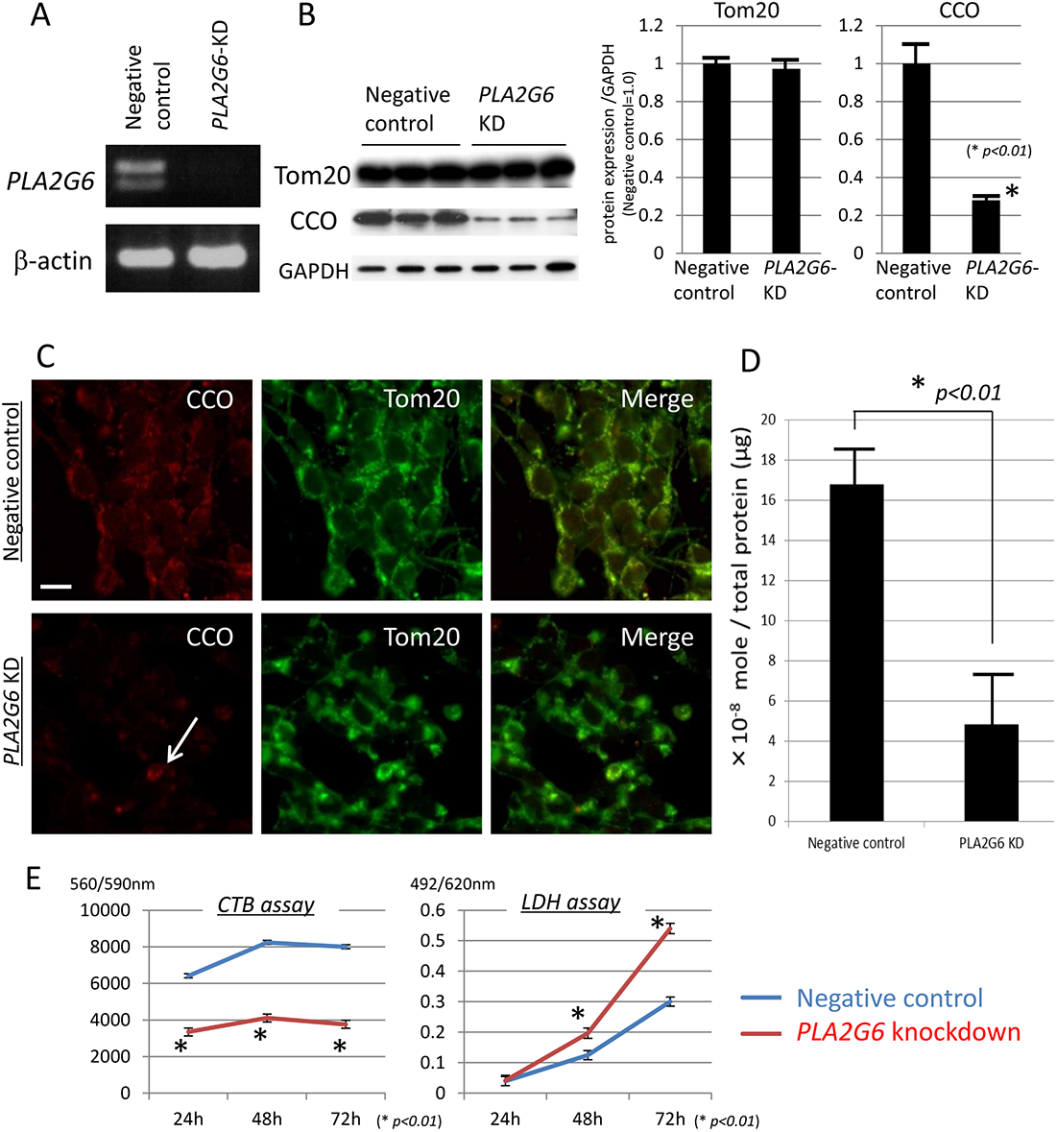
Examination of mitochondrial function and cell viability in *PLA2G6*-KD cells. Each experiment was performed at least three times. (A) Generation of *PLA2G6-*KD gene SH-SY5Y cells. Optimal KD efficacy is confirmed by Reverse transcriptase polymerase chain reaction (RT-PCR). (B, C) Western blotting analyses (B) and immunocytochemistry (C) for Tom20 (the marker for the mitochondrial outer membrane) and CCO (the marker of the mitochondrial inner membrane) in *PLA2G6*-KD cells. (B) Expression levels of CCO are decreased in *PLA2G6*-KD cells compared with those of SH-SY5Y cells treated with negative control siRNA with statistical significance (*p < 0.*01*, Wilcoxon's rank sum test), whereas there is no significant difference in expression levels of Tom20 between *PLA2G6*-KD cells and the negative control. Data are presented as the ratio of CCO or Tom20 to GAPDH (negative control = 1.0), respectively. The experiment was performed three times. Each bar represents the mean ± SD. (C) Immunocytochemistry for CCO (red) and Tom20 (green). Expression levels of CCO are reduced in *PLA2G6*-KD cells, and only a few cells are positive for CCO (white arrow). Scale bar represents 10 μm for each panel. (D) The amount of ATP in each cell is measured by a Kinshiro ATP luminescence kit. The ATP generation per total proteins is significantly reduced in *PLA2G6*-KD cells in comparison with negative control siRNA-transfected SH-SY5Y cells (*p < 0.*01*, Wilcoxon's rank sum test). The experiment was performed three times. Each bar represents the mean ± SD. (E) Knockdown of *PLA2G6* gene significantly reduces cell viability in both CTB and LDH assays (*p < 0.*01*, compared with negative control, Wilcoxon's rank sum test). The experiment was performed five times and the averages of the results are shown.

We have already established cultures of *PLA2G6-*KD SH-SY5Y human neuroblastoma cells (Fig 7A). We have also demonstrated that the expression level of CCO, a marker for mitochondrial inner membrane, was significantly reduced in *PLA2G6*-KD cells in comparison with SH-SY5Y cells transfected with negative control siRNA by both Western blotting (*p < 0*.*01*, Wilcoxon's rank sum test, Fig 7B) and immunocytochemistry (Fig 7C). On the other hand, the expression level of Tom20, a marker for mitochondrial outer membrane, was not decreased in *PLA2G6*-KD cells (Fig 7B and 7C). In our previous study, we detected the presence of abnormal mitochondria with degenerated inner membranes, which were strongly positive for Tom20 and negative for CCO by immunohistochemistry, in the CNS of iPLA_2_β-KO mice [28]. These results indicated that mitochondrial inner membrane was mainly degenerated in *PLA2G6*-KD cells similar to iPLA_2_β-KO mice. Mitochondrial ATP production significantly decreased in *PLA2G6*-KD cells in comparison with the negative control (Fig 7D). CTB and LDH assays showed a significant reduction in cell viability in *PLA2G6*-KD cells, particularly 48 h after transfection, compared with negative control (*p < 0*.*01*, Wilcoxon's rank sum test, Fig 7E). The reduction of resorufin in the CTB assay could be induced by mitochondrial dysfunction in *PLA2G6*-KD cells because it was reported that resazurin can be converted to resorufin by mitochondrial enzymes [29]. These results suggest that knockdown of *PLA2G6* could cause mitochondrial dysfunction *in vitro*.

**Fig 8:**
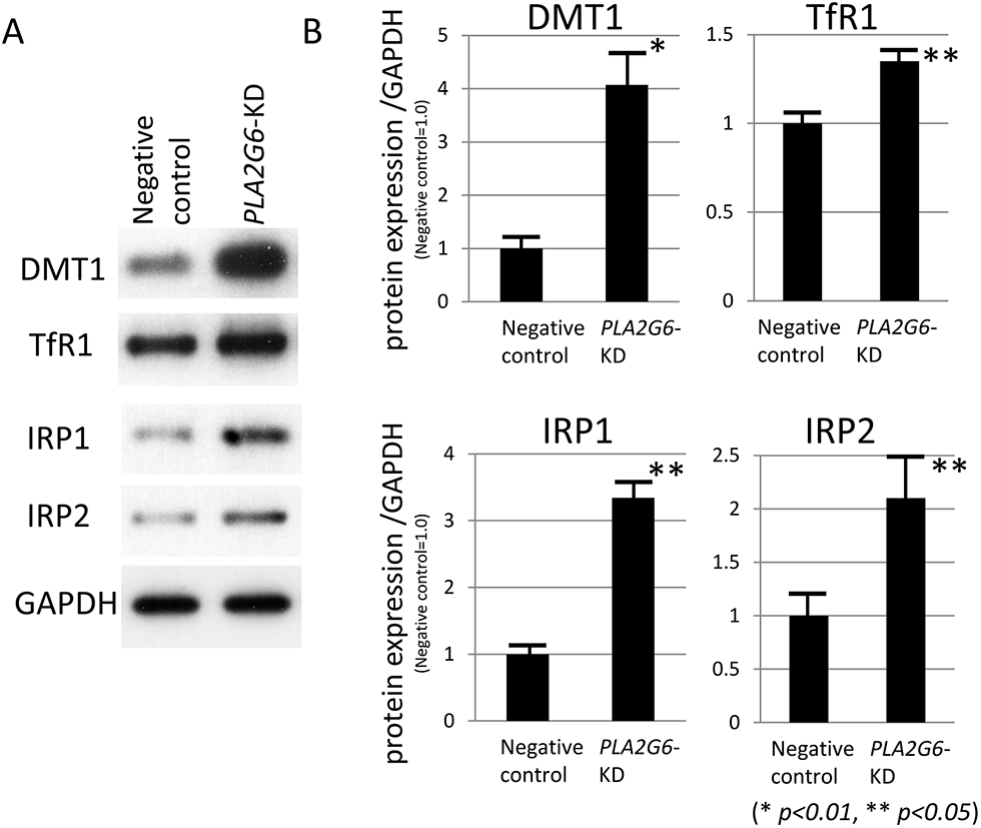
Western blotting analyses of the molecules involved in cellular iron homeostasis in *PLA2G6*-KD cells. Each experiment was performed three times and the averages of the results are shown. (A, B) Expression levels of DMT1 + IRE, TfR1, IRP1, and IRP2 are significantly increased in *PLA2G6*-KD cells compared with SH-SY5Y cells treated with negative control siRNA with statistical significance (*p < 0.*01*, **p < 0.*05*, Wilcoxon's rank sum test), respectively. (B) Data are presented as the ratio of DMT1, TfR1, IRP1 or IRP2 to GAPDH (negative control = 1.0), respectively. Each bar represented the mean ± SD of four independent experiments.

Next, we examined the expression levels of molecules that are involved in iron homeostasis in *PLA2G6*-KD cells. On Western blotting analysis, the expression levels of DMT1 + IRE, TfR1, IRP1, and IRP2 were all increased in *PLA2G6*-KD cells compared with those in cells treated with negative control siRNA (Fig 8A); results were statistically significant (*p < 0*.*01* or *p < 0*.*05*, Wilcoxon's rank sum test, Fig 8B) and were identical to those observed in the examination of mouse brain (Figs 3 and 4).

## Discussion

In the present study, we demonstrated age-dependent and prominent iron accumulation in the CNS of iPLA_2_β-KO mice. Iron accumulation was particularly prominent in the SN of iPLA_2_β-KO mice compared with age-matched WT control mice. The increased expressions of the molecules involved in iron homeostasis, particularly DMT1 and IRP2, were also demonstrated in the brains of aged iPLA_2_β-KO mice as well as in *PLA2G6*-KD SH-SY5Y human neuroblastoma cells. This is the first reported study to demonstrate iron accumulation and the up-regulation of iron transporters in the brain during the deficiency of iPLA_2_β.

Iron accumulates during aging in normal brains [30] and in greater amounts during several neurologic disorders such as Parkinson’s disease (PD [31]) and Alzheimer’s disease (AD [30]). Previous studies have shown that the distribution of iron in the brain is uneven; the highest concentrations of iron are observed in the SNpr, ST, and Gp [31]. Our results showing that significant iron accumulation was observed in the SN, ST, and Gp would be compatible with these distributions of iron. Most of the iron depositions were observed in oligodendrocytes in the brain [32, 33]. The only known function of oligodendrocytes is myelin production, and both a direct and indirect relationship exists between iron acquisition and myelin production [33]. However, the mechanism of iron uptake in oligodendrocytes remains unknown [17].

In this study, we have demonstrated for the first time that prominent brain iron accumulation is observed in aged iPLA_2_β-KO mice. This accumulation was examined by pathological examination, and was similar to that of human patients with INAD and NBIA2 [12, 34]. Iron accumulation in the brains of iPLA_2_β-KO mice was most significant in special areas such as SN, Gp, and ST, and is identical with human patients with NBIA2 [12, 34]. These results suggest that this animal model would be very useful to examine the mechanisms underlying iron-induced neurodegeneration. Previously, Malik et al reported that Prussian blue staining for pathological iron accumulation did not provide evidence of iron accumulation in Gp and SN of iPLA_2_β-KO mice [15]. It is well known that the sensitivity of iron staining is drastically enhanced by Perl’s method with DAB treatment [26]. Our results suggest that Prussian blue staining may be insufficient to visualize the iron deposition in iPLA_2_β-KO mice.

We also showed an increase of 4-HNE, the major end products of lipid peroxides, in the ST and SN of aged iPLA_2_β-KO mice. Iron is known to catalyze the formation of ROS such as hydroxyl radicals and initiation or enhancement of lipid peroxidation by reacting with hydrogen peroxide (H_2_O_2_) via the Fenton reaction [21]. Accumulation of iron could lead to the formation of 4-HNE through the Fenton reaction, and the increase of 4-HNE was observed mainly in the white matter (Fig 5B–5D) probably because brain iron accumulation was frequently observed in oligodendrocytes in iPLA_2_β-KO mice. It was reported that a chronic increase in IRP2, which enhanced iron uptake, also induced mitochondrial oxidative insults and accelerated neurodegeneration in mice [35]. In this study, our results at 56 weeks showed no evidence of dopaminergic cell death despite detection of prominent iron accumulation in iPLA_2_β-KO mice relative to WT mice. These results indicated that iron accumulation preceded neuronal loss in iPLA_2_β-KO mice. Generation of ROS through Fenton reaction might cause neurodegeneration in specific regions of the brain such as SN after 56 weeks in iPLA_2_β-KO mice.

The first step in blood to brain transport of iron is receptor-mediated endocytosis of transferrin by BVECs. Once inside the brain, iron is transported to the neurons via TfR1 and/or DMT1 [17, 18]. Recently, DMT1 has been emphasized as a molecule that plays an important role in iron uptake in neurodegenerative disorders such as PD. A lot of studies have reported that DMT1 is upregulated in patients with PD [36], animal models [36, 37, 38], and in vitro models [37, 38, 39]. In the present study, we demonstrated that DMT1 was prominently upregulated in the brains of iPLA_2_β-KO mice compared with those of age-matched WT mice. Although TfR1 was also upregulated in some brain areas such as ST in iPLA_2_β-KO mice, our results suggest that DMT1 could play more important role than TfR1 in iron uptake in deficiency of iPLA_2_β. Moreover, downregulation of FPN1, which is a transmembrane protein that transports iron from the inside of a cell to the outside of it, was also found in iPLA_2_β-KO mice. The same results were observed in PD models [38].

We also demonstrated the upregulation of IRP1 and 2 in aged iPLA_2_β-KO mice compared with age-matched WT mice. Among them, IRP2 showed a greater increase in iPLA_2_β-KO mice. IRPs could sense intracellular iron status and participate in the maintenance of cellular iron homeostasis [19]. It was reported that IRP2 is sensitive to iron status and can compensate for the loss of IRP1 by increasing its binding activity [40]. IRP2 is supposed to play a major role in iron homeostasis in the CNS as indicated by the fact that genetic ablation of IRP2 causes a progressive neurodegeneration in mice [41]. These results suggest that increased IRP2 would bind to the IRE in the 3′-UTR of DMT1 + IRE and then increase their stability in iPLA_2_β-KO mice.

The question of why IRPs were activated in the presence of iPLA_2_β deficiency was asked. Mitochondrial dysfunction may be one of the answers to this question. In the previous report, we demonstrated by ultrastructural analysis that the mitochondrial inner membrane was degenerated in iPLA_2_β-KO mice since an early age; the mitochondrial membrane degeneration had been induced by insufficient remodeling and accumulation of cardiolipin (CL) [28]. Similarly, cytochrome oxidase activity (COX, complex IV) was reported to be reduced in respiratory chain enzyme analysis in one human case with INAD [42]. Here we showed that an *in vitro* deficiency of iPLA_2_β led to the degeneration of the mitochondrial inner membrane and reduction of CCO expression. Mitochondrial inner membranes could be easily affected because of deficiency of iPLA_2_β, which can hydrolyze peroxidized fatty acids to repair the membrane phospholipids oxidized by ROS [43, 44]. 4-HNE is considered to be an oxidized secondary product of lipids, including CL [45], and would reflect mitochondrial dysfunction [46]. The increase of 4-HNE observed in iPLA_2_β-KO mice may be induced by iron accumulation combined with mitochondrial dysfunction.

Furthermore, we demonstrated that the genetic ablation of iPLA_2_β reduced ATP production in human neuronal cell line mitochondria. Recently, it has been reported that loss of normal *PLA2G6* gene activity leads to lipid peroxidation, mitochondrial dysfunction, such as reduced ATP synthesis, and subsequent mitochondrial membrane abnormalities in an iPLA_2_β-KO fly model and also in fibroblasts from patients with *PLA2G6* mutations [47]. Similar to MPTP (1-methyl-4-phenyl-1,2,3,6-tetrahydropyridine) -associated inhibition of mitochondrial complex I, mitochondrial dysfunction in iPLA_2_β deficiency could cause both a rise in ROS and a depletion of ATP; these two events have been shown to augment the binding activity of IRPs [22, 23] that control the expression of DMT1 + IRE and/or TfR1 and the subsequent increase in iron content [19].

Recently, it has been reported that patients with adult onset, L-dopa responsive, dystonia-parkinsonism, known as PARK14, were found to have mutations in the *PLA2G6* gene [48]. In previous reports, most of these cases showed the absence of iron depositions on MRI [48, 49]. A possible explanation for this is a difference in catalytic activity of iPLA_2_β enzymes between the different disease phenotypes, INAD/NBIA and dystonia-parkinsonism [50]. However, our results suggest that PD and PLAN could have some common pathway for iron accumulation and neurodegeneration in some brain regions such as SN and ST.

In conclusion, deficiency of iPLA_2_β could cause activation of IRP2 and may upregulate DMT1 at the post-transcriptional level, which may be associated with mitochondrial dysfunction, leading to subsequent iron accumulation in the brain. Our results suggest that PLAN and PD may share a common pathway for brain iron accumulation. Using this animal model, further investigations on the mechanisms of iron-associated neurodegeneration and therapeutic targets in NBIA, probably also on PD, could be performed in the future.

## Supporting Information

S1 Fig Double staining for glial cell markers and iron staining in iPLA_2_β-KO mice. (A, B) Perl’s DAB enhanced staining (brown) and immunohistochemistry for GFAP (A, red) or Iba-1 (B, red) of iPLA_2_β-KO mice at 100 weeks. Glial cells positive for iron staining (small arrows in A and B) are negative for GFAP (A) or Iba-1 (B). GFAP-positive astrocytes (arrowheads in A) and Iba-1-positive microglial cells (arrowheads in B) are observed. Scale bar in (A) represents 50 μm in (A) and (B).
(TIF)

S2 Fig Iron staining of the ST and Gp of WT mice at 100 weeks. (A, B) Perl’s DAB enhanced staining of the ST (A) and Gp (B) of WT mice at 100 weeks. Scale bar in (A) represents 50 μm in (A) and (B) (high power field, HPF).
(TIF)

S3 Fig Iron accumulation in neurons of iPLA_2_β-KO mice shown by DAB enhancement of Perl’s staining. (A‒C) Perl’s DAB enhanced staining of the cerebellum (A, B) and hippocampus (C) of iPLA_2_β-KO mice at 100 weeks. Iron depositions in neurons are frequently observed in the dentate nucleus (A) and Purkinje cells (B, arrows) of the cerebellum of iPLA_2_β-KO mice at 100 weeks. The inset in (A) is a high-magnification view of the neuron with iron deposition. Scale bar in (A) represents 50 μm in (A), 20 μm in (B) and (C), respectively.
(TIF)

S4 Fig Western blotting analysis of FPN1 in iPLA_2_β-KO mice at 100 weeks. (A) Western blotting was applied to detect the expression of FPN1 in iPLA_2_β-KO mice and WT mice at 100 weeks. (B) Statistical analysis. Data are presented as the ratio of FPN1 to GAPDH (WT mice = 1.0). Each bar represents the mean ± SD. *p < 0.*05*, Wilcoxon's rank-sum test.
(TIF)
