# Supplementary figures and images for: Deficiency of Calcium-Independent Phospholipase A2 Beta Induces Brain Iron Accumulation through Upregulation of Divalent Metal Transporter 1
Source: PLoS One. 2015 Oct 27;10(10):e0141629. doi: 10.1371/journal.pone.0141629 (PMC4624760; doi:10.1371/journal.pone.0141629)

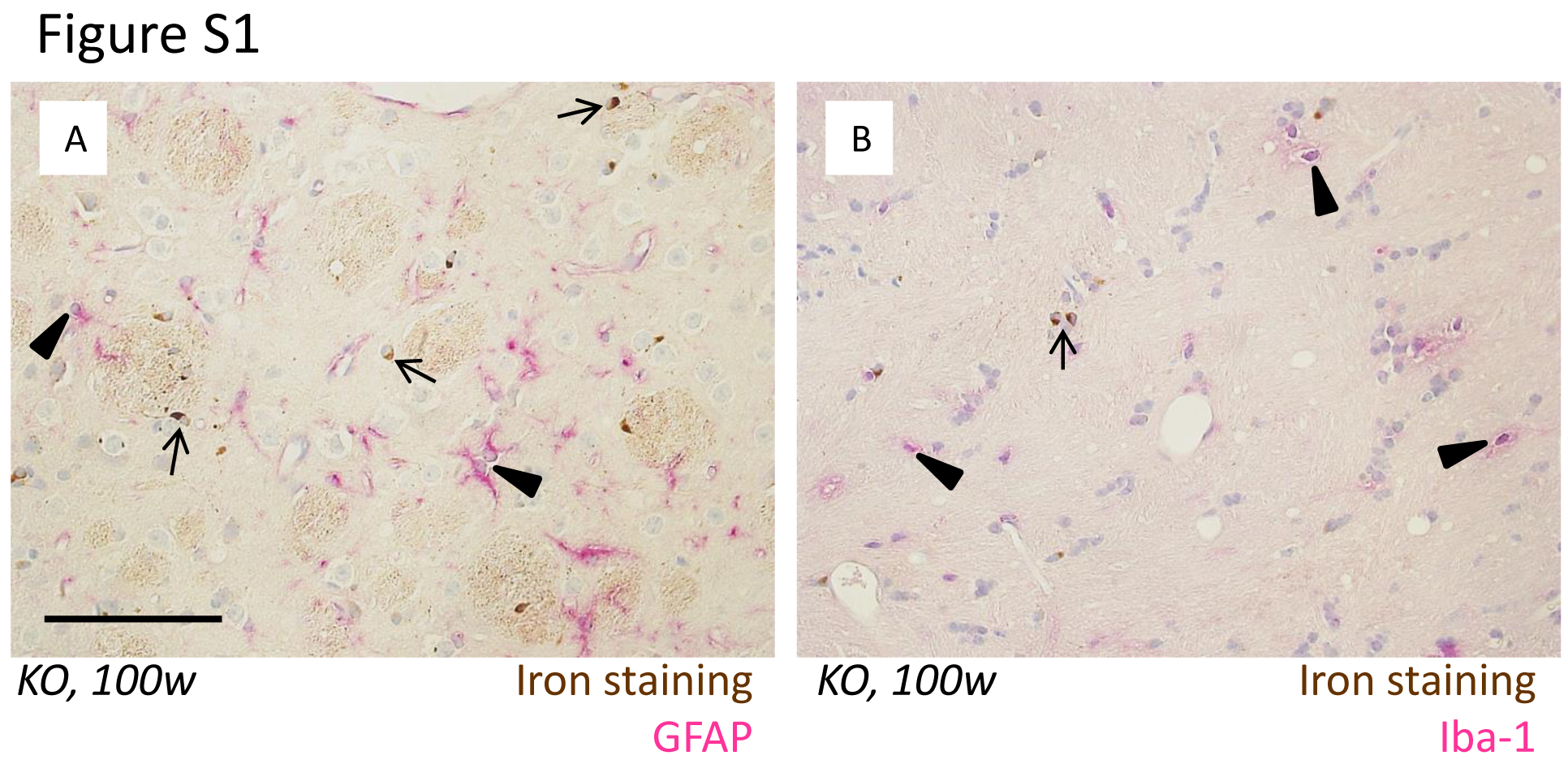

Supplement: S1 Fig — (A, B) Perl’s DAB enhanced staining (brown) and immunohistochemistry for GFAP (A, red) or Iba-1 (B, red) of iPLA2β-KO mice at 100 weeks. Glial cells positive for iron staining (small arrows in A and B) are negative for GFAP (A) or Iba-1 (B). GFAP-positive astrocytes (arrowheads in A) and Iba-1-positive microglial cells (arrowheads in B) are observed. Scale bar in (A) represents 50 μm in (A) and (B). (TIF) [file pone.0141629.s001.tif]

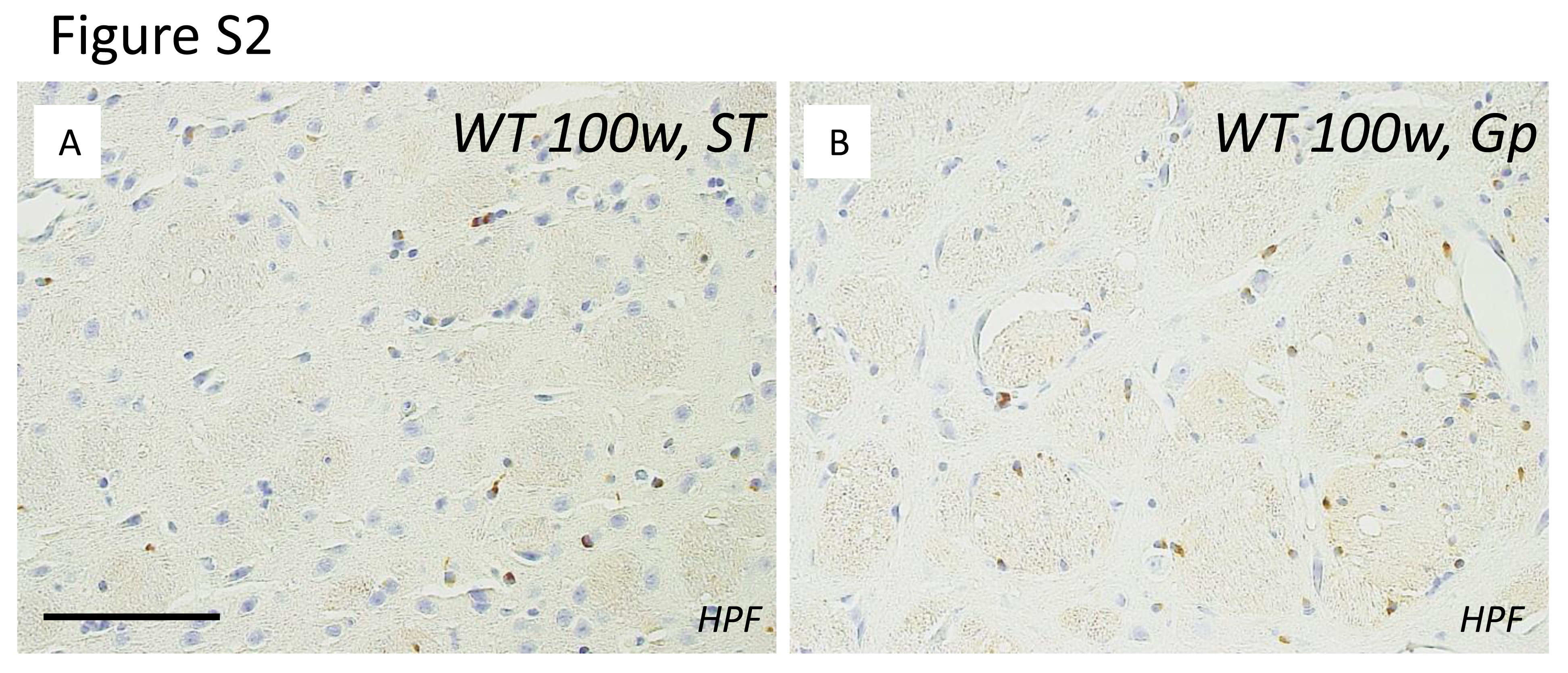

Supplement: S2 Fig — (A, B) Perl’s DAB enhanced staining of the ST (A) and Gp (B) of WT mice at 100 weeks. Scale bar in (A) represents 50 μm in (A) and (B) (high power field, HPF). (TIF) [file pone.0141629.s002.tif]

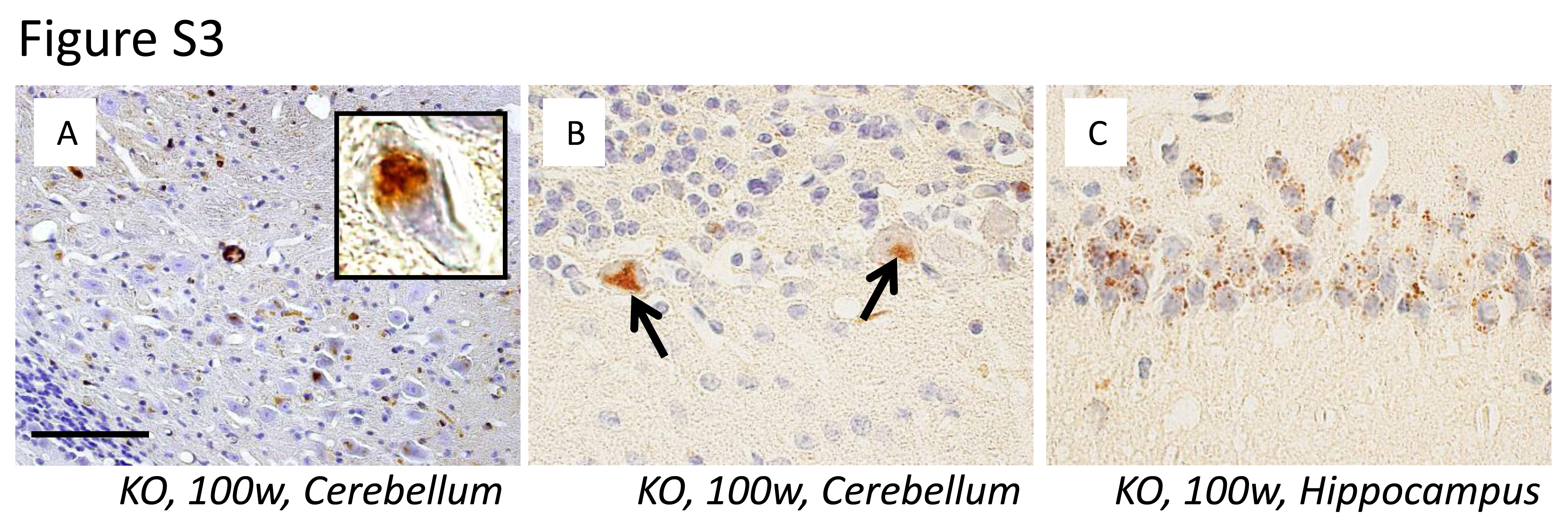

Supplement: S3 Fig — (A‒C) Perl’s DAB enhanced staining of the cerebellum (A, B) and hippocampus (C) of iPLA2β-KO mice at 100 weeks. Iron depositions in neurons are frequently observed in the dentate nucleus (A) and Purkinje cells (B, arrows) of the cerebellum of iPLA2β-KO mice at 100 weeks. The inset in (A) is a high-magnification view of the neuron with iron deposition. Scale bar in (A) represents 50 μm in (A), 20 μm in (B) and (C), respectively. (TIF) [file pone.0141629.s003.tif]

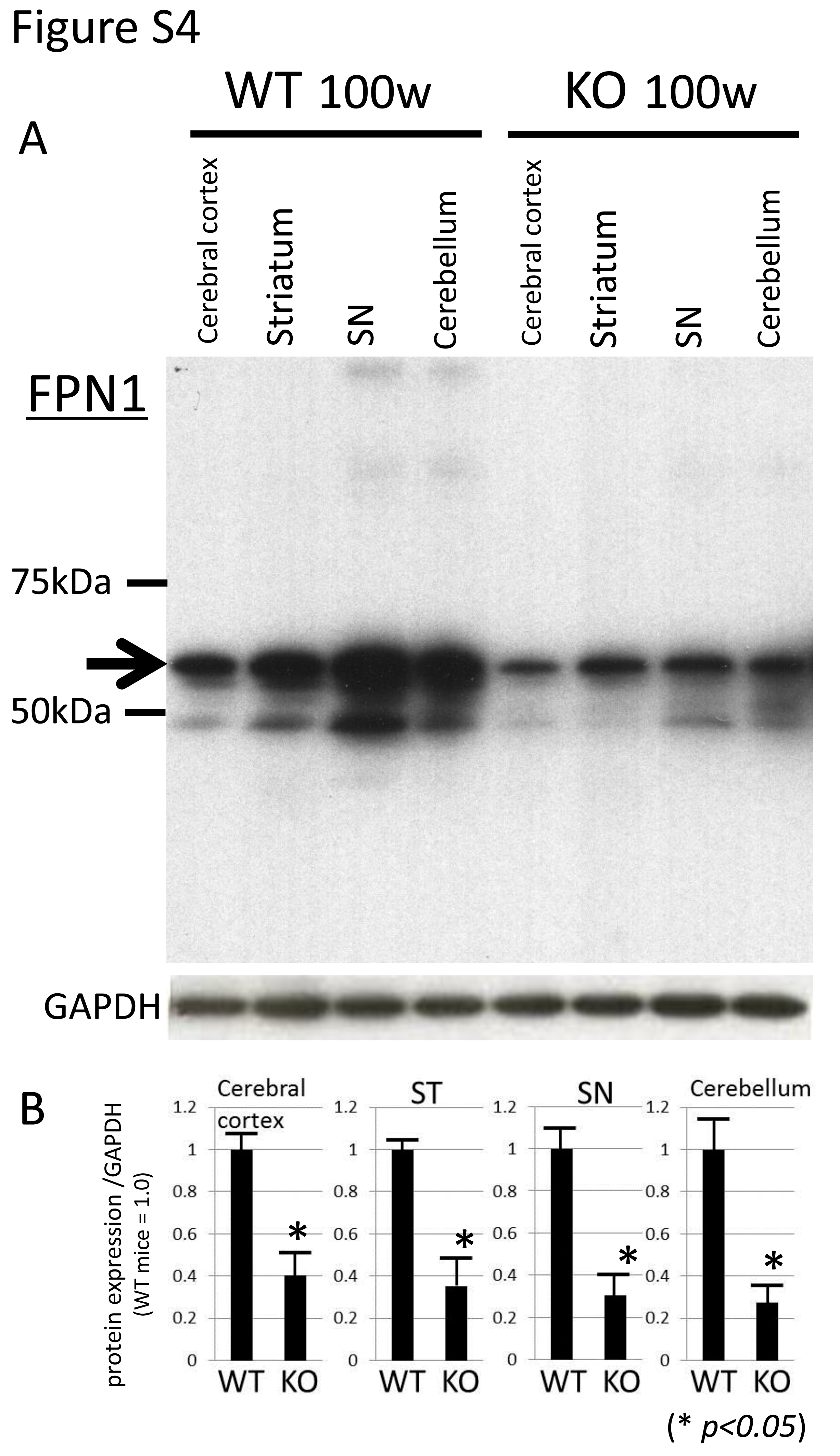

Supplement: S4 Fig — (A) Western blotting was applied to detect the expression of FPN1 in iPLA2β-KO mice and WT mice at 100 weeks. (B) Statistical analysis. Data are presented as the ratio of FPN1 to GAPDH (WT mice = 1.0). Each bar represents the mean ± SD. *p < 0.05, Wilcoxon's rank-sum test. (TIF) [file pone.0141629.s004.tif]
